# CNVs are associated with genomic architecture in a songbird

**DOI:** 10.1186/s12864-018-4577-1

**Published:** 2018-03-13

**Authors:** Vinicius H. da Silva, Veronika N. Laine, Mirte Bosse, Kees van Oers, Bert Dibbits, Marcel E. Visser, Richard P. M. A. Crooijmans, Martien A. M. Groenen

**Affiliations:** 1 Animal Breeding and Genomics Centre, Wageningen University & Research, Droevendaalsesteeg 1, Wageningen, 6708PB The Netherlands; 2 Netherlands Institute of Ecology (NIOO-KNAW), Droevendaalsesteeg 10, Wageningen, 6708PB The Netherlands; 3 Swedish University of Agricultural Sciences (SLU), Ulls väg 26, Uppsala, 750 07 Sweden

**Keywords:** *Parus major*, Genetic variation, Inheritance, Duplication, Recombination

## Abstract

**Background:**

Understanding variation in genome structure is essential to understand phenotypic differences within populations and the evolutionary history of species. A promising form of this structural variation is copy number variation (CNV). CNVs can be generated by different recombination mechanisms, such as non-allelic homologous recombination, that rely on specific characteristics of the genome architecture. These structural variants can therefore be more abundant at particular genes ultimately leading to variation in phenotypes under selection. Detailed characterization of CNVs therefore can reveal evolutionary footprints of selection and provide insight in their contribution to phenotypic variation in wild populations.

**Results:**

Here we use genotypic data from a long-term population of great tits (*Parus major*), a widely studied passerine bird in ecology and evolution, to detect CNVs and identify genomic features prevailing within these regions. We used allele intensities and frequencies from high-density SNP array data from 2,175 birds. We detected 41,029 CNVs concatenated into 8,008 distinct CNV regions (CNVRs). We successfully validated 93.75% of the CNVs tested by qPCR, which were sampled at different frequencies and sizes. A mother-daughter family structure allowed for the evaluation of the inheritance of a number of these CNVs. Thereby, only CNVs with 40 probes or more display segregation in accordance with Mendelian inheritance, suggesting a high rate of false negative calls for smaller CNVs. As CNVRs are a coarse-grained map of CNV loci, we also inferred the frequency of coincident CNV start and end breakpoints. We observed frequency-dependent enrichment of these breakpoints at homologous regions, CpG sites and AT-rich intervals. A gene ontology enrichment analyses showed that CNVs are enriched in genes underpinning neural, cardiac and ion transport pathways.

**Conclusion:**

Great tit CNVs are present in almost half of the genes and prominent at repetitive-homologous and regulatory regions. Although overlapping genes under selection, the high number of false negatives make neutrality or association tests on CNVs detected here difficult. Therefore, CNVs should be further addressed in the light of their false negative rate and architecture to improve the comprehension of their association with phenotypes and evolutionary history.

**Electronic supplementary material:**

The online version of this article (10.1186/s12864-018-4577-1) contains supplementary material, which is available to authorized users.

## Background

Genetic variants in the genome have been selected over the course of evolution based on their adaptive value under changing environmental conditions but are also affected by random drift [1]. These variants range from single nucleotide changes to complex rearrangements in structure [2], which modulate gene expression [3–5] leading to ample phenotypic variation in wild populations [6–8]. Structural variants show different degrees of complexity, and include copy number variations (CNVs), inversions, insertions, translocations, fissions and fusions [9, 10]. A better understanding of these structural variants is essential for detecting important genomic features under selection and their association with phenotypes. In fact, CNVs are known to be major mutations that encompasses more nucleotides than single nucleotide polymorphisms (SNPs) [11] and underlie differences within populations and between closely related species such as human and chimpanzee [12].

Although complex rearrangements in the genome which involves combined events like inversions and translocations can be technically challenging and costly to fully characterize [13], CNVs are more easily assessed and be an indication of complex variants [14]. Moreover, CNVs are the raw material for gene family expansion and diversification [15], which ultimately lead to repetitive regions that have an important role in evolutionary breakpoints [16]. CNVs are usually defined as genomic intervals larger than 1 kilobase (kb) containing deletions or duplications, which can be studied using widely available SNP arrays [17]. Despite their limited resolution, these SNP arrays are cost effective for studies in large populations [18] and CNVs can be uncovered by signal variability and heterozygosity level in overlapping SNP probes [17].

Species-specific SNP arrays have been used extensively to study CNVs and their association with phenotypes and evolutionary history, in domestic animals [19, 20], humans [12, 21] and natural populations [22]. In mammals, CNVs has been associated with production traits [23] and pathogen resistance [24]. Deletions or duplications of genes underpinning inflammatory response and cell proliferation are involved in the phenotypic differentiation of humans and chimpanzees [12]. An interesting example of phenotypic variation as a result of CNV is the pea-comb phenotype in chicken which is caused by a CNV in intron 1 of SRY-Box 5 (*SOX5*, [25]). Interestingly, the number of repeats quantitatively affects this phenotype when in heterozygous state [26]. Although CNVs are increasingly recognized as source of phenotypic variation, other aspects of CNVs as their inheritance, genomic distribution and rate of false positive or negatives lacks further investigation in large populations.

CNVs usually follow a Mendelian inheritance pattern [27], but also de novo mutations have been shown to be functionally relevant and to be associated with a number of diseases [28]. Structural rearrangements, like CNVs, result from a number of distinct recombination mechanisms (for a review see [29]). Such mechanisms like non-allelic homologous recombination or break induced replication prevails at regions in the genome exhibiting specific architecture like segmental duplications and common fragile sites, respectively. Moreover, structural mutability is associated with hypomethylation [30, 31] and CpG islands and transcription start and end sites have been shown to be associated with high recombination rates in birds [32].

We identified and studied CNVs in a natural population of great tits (*Parus major*). The great tit is a widely studied passerine bird species in ecology that, in the past decades, has provided important insights into speciation [33], phenology [34–36], behavior [37, 38] and microevolution [39]. After completion of the great tit genome sequence [40], a customized high density 650k SNP array was developed enabling more detailed genomic studies in this species. We present a CNV analysis in the great tit genome using intensities and allele frequencies from this SNP array. We annotated functional features, accessed mother-daughter inheritance and characterized the genomic architecture underlying different molecular mechanisms, which in turn are known to give rise to different CNV classes. Our study lays the foundations for future studies on complex genetic variants in this population, to better understand genetic variation under global warming and association with shifting seasonal phenotypes.

## Results

### CNV identification, frequency assignment and inheritance

We performed a CNV analysis in great tit genomes using a high density SNP array intensities and allele frequencies from 2,077 females and 98 males. After quality control, 41,029 CNVs were identified which were subsequently merged into 8,008 distinct CNV regions (CNVRs, [Additional file 1]).

The CNVRs cover 28.09% (259.50 millions of base pairs - Mb) of the great tit autosomes. The relative coverage by CNVRs for the different chromosomes ranged from 20.18% for chromosome 14 to 89.30% for chromosome 25LG2. The absolute genomic length overlapped by CNVRs varied from 0.36 Mb for chromosome LGE22 to 40.06 Mb for chromosome 2. The CNVRs had variable sizes ranging from 1.01 kb to 2.83 Mb with a mean size of 32.40 kb. The number of birds with CNVs mapped onto a given CNVR ranged from 1 (0.04%) to 623 (28.63%) of the 2,175 birds analyzed. We found 263 CNVRs to occur in more than 1% of the population (≥ 21 birds) and denote them as ‘91polymorphic CNVRs’ as previously suggested [41].

**Fig. 1:**
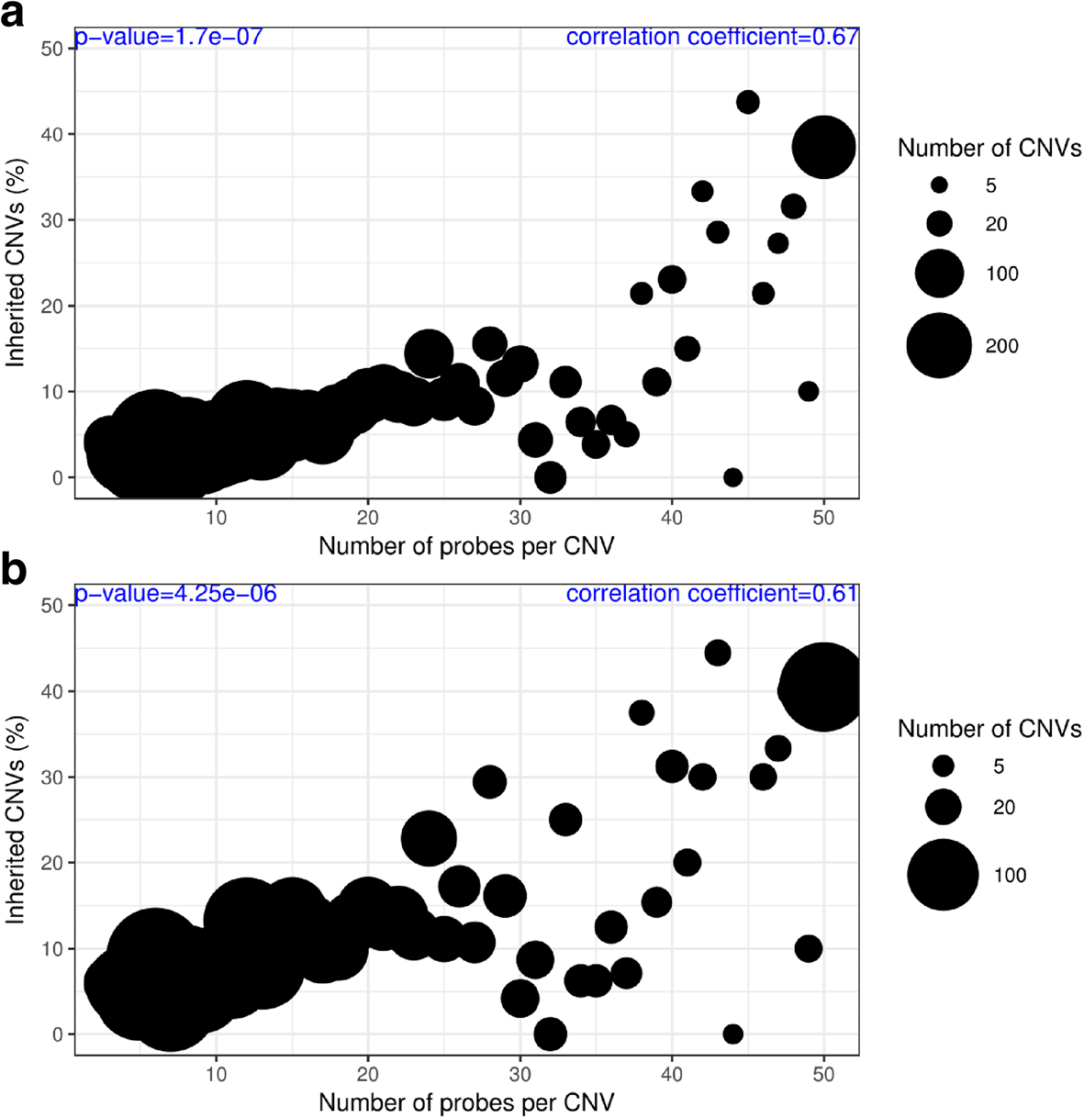
CNV inheritance in mother-daughter family structure. We inferred the percentage of CNVs in mothers overlapping CNVs at the same state (gain or loss) in their respective daughters. The *x*-axis indicates distinct groups of CNVs which were classified based on the number of SNP probes supporting each of them. CNVs supported by 50 SNP probes or more are grouped together. In the *y*-axis the percentage of inherited CNVs represents the ratio between all CNVs and inherited ones in each probe group. The number of CNVs per group is reflected by the dot size. **a**: All CNVRs. **b**: Polymorphic CNVRs (≥ 21 birds, at least 1% of the population with CNVs identified)

To investigate CNV inheritance, we used a mother-daughter structure available for 381 mothers and their 625 daughters in this population. We found 460 CNV calls that overlap at least 1 base pair (bp) in the same state (gain or loss) between a mother and at least one of her respective daughters, representing only 6.83% of all 6,733 CNVs identified in the mothers. Thereafter, we classified all CNVs in mothers depending on the number of probes by CNV and found a positive correlation between probe number and inheritance ratio (Pearson’s correlation coefficient = 0.62 and *p*-value ≈1.68e−7). Considering an expected Mendelian inheritance of 50% (all sires in normal state), only CNVs supported by 40 probes or more reach this Mendelian expectancy (for most of the probe groups, Fig. 1a). Also, CNVs within polymorphic CNVRs showed higher inheritance ratios (367 out of 3,035, 12.09%) but comparable positive correlation with probe number (Pearson’s correlation coefficient = 0.60 and *p*-value ≈ 4.254e-06, Fig. 1b).

**Fig. 2:**
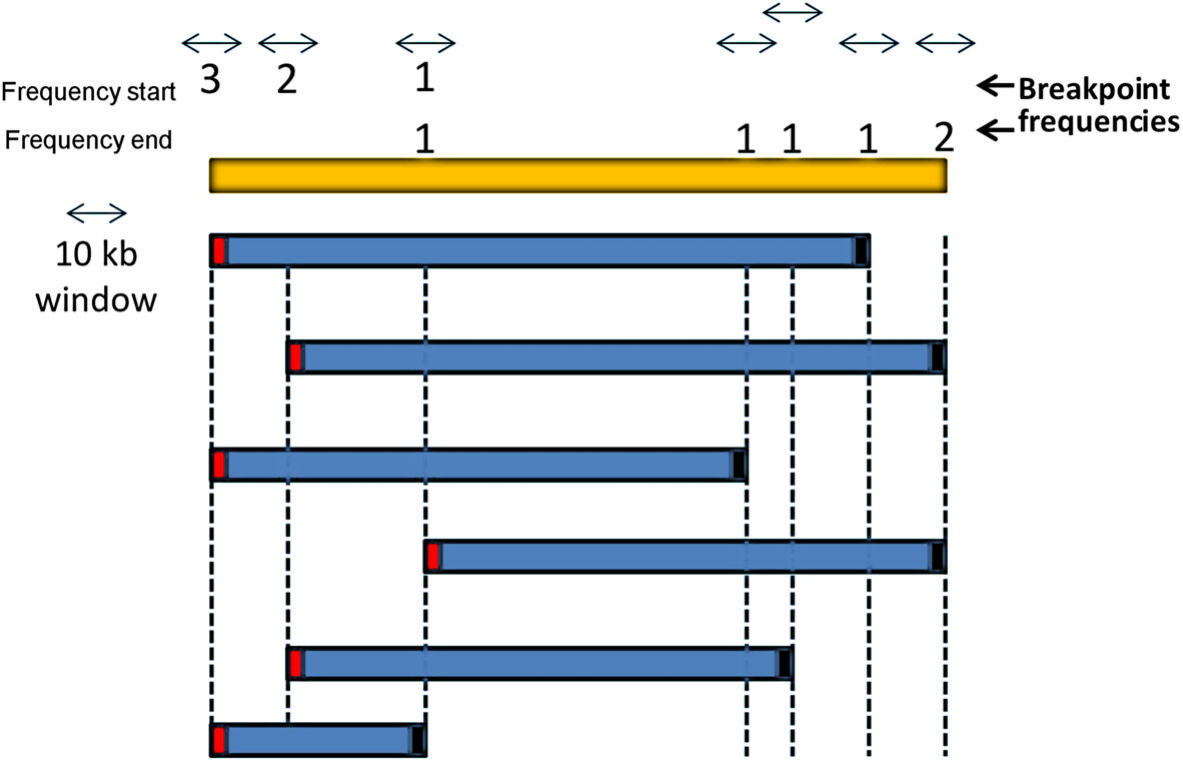
CNVR example and the strategy to estimate the frequency of CNVs which are sharing breakpoints. The frequency for a given genomic interval is given by the number of CNVs starting or ending at certain SNP probes. All the windows around the breakpoints have 10 kb and may have one frequency for the common start positions and one for the end positions

Breakpoint variability of overlapping CNVs can unravel molecular mechanisms in their formation and inheritance patterns, which in turn rely on specific patterns in genome architecture [29]. However, there is an unavoidable technical bias in genomic breakpoints of CNVs based on SNP probe intensities [11, 42], making it challenging to estimate the frequency of CNV loci. To avoid coarse-grained CNVR breakpoints, which can harbor several CNVs with distinct breakpoints, we tried to improve our description of the breakpoint variability using the number of CNVs sharing the same start or end positions (Fig. 2). We extended each of these breakpoints by 5 kb up and downstream to establish genomic windows of 10 kb (CNV breakpoint windows). This resulted in 45,372 breakpoint windows identified in 1 to 355 birds. The total of these windows represents 254.14 Mb of the genome, which the large majority (224.38 Mb) reflects rare events (frequency = 1, CNV breakpoint windows and their corresponding frequencies can be found at [Additional file 2]).

### Copy number inference by quantitative PCR

To obtain insight in the false discovery rate of our method to identify CNVs, we validated 16 CNVs in our great tit population using quantitative PCR (qPCR). We selected 6 rare and 10 frequent CNV calls based on CNV incidence, size and state. Concerning incidence, we selected CNVs identified in only one bird, those present in two and those present in four to five birds (all with exactly the same breakpoints). Within each frequency class we tried to choose different sizes of events. Concerning state, in each frequency class we ensured the inclusion of at least one CNV belonging to each of the most common states (1n and 3n). The size of the CNVs chosen for validation ranged from 1.06 to 77.12 kb, and are located within CNVRs ranging from 1.06 to 494.36 kb. The number of SNPs supporting these CNVs ranges from 3 to 19. The gain or loss of specific genomic intervals, detected by PennCNV, was confirmed by qPCR for 15 of these 16 CNVs (93.75%). However, we observed discrepancies in the copy number based on PennCNV and qPCR. Considering exactly the same state (i.e. copy number between one and four), 9 out of the 16 CNVs (56.25%) showed the same number of copies using these two methods [Additional file 3].

### Repetitive and functional intervals in CNVs

We evaluated five different sequence features in the great tit genome for their overlap with CNV breakpoint windows: (I) Homologous regions, (II) Interspersed repeats and low complexity DNA sequences, (III) CpG sites, (IV) Transcription start sites (TSSs) and (V) AT-rich regions.

**Fig. 3:**
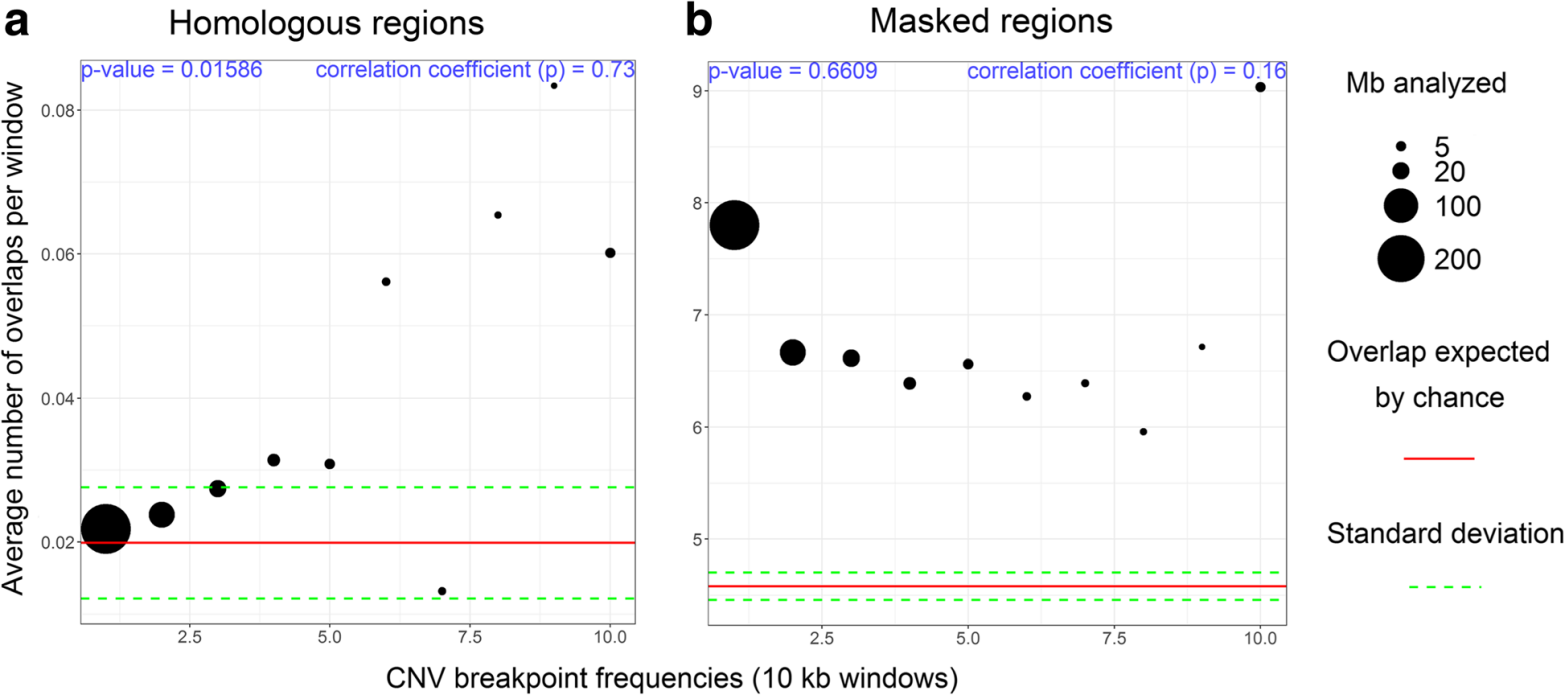
Overlap of CNV breakpoints with repetitive regions in the genome. CNV breakpoints with 10 in frequency or more are grouped together. **a**: Homologous regions with more than 90% in similarity and 1 kb. **b**: Masked regions as retroelements, RNA-related regions, DNA transposons and *in-tandem* repeats

**Fig. 4:**
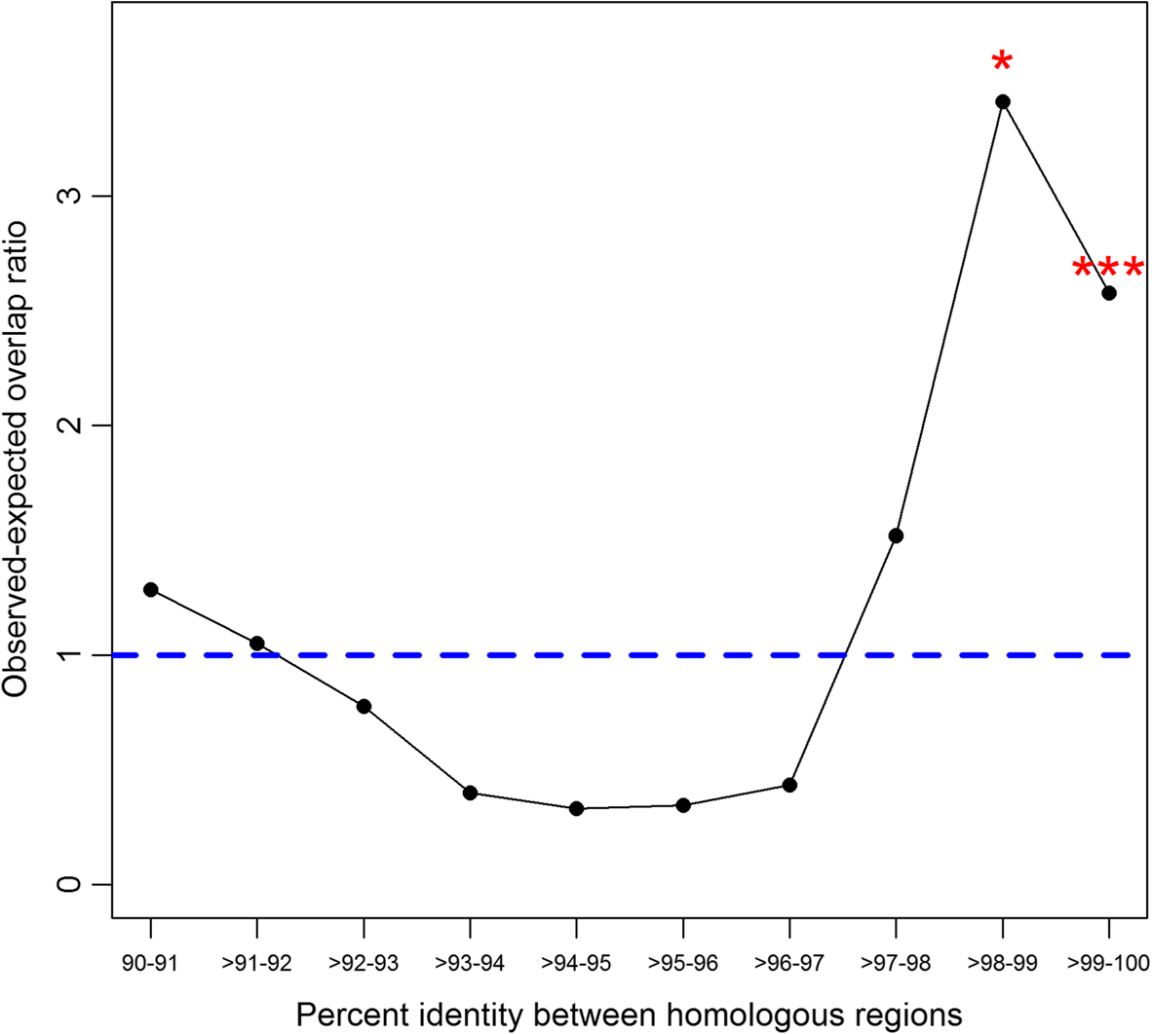
Colocalization of CNV breakpoints (10 kb windows with ≥ 4 in frequency) and homologous regions binned by sequence identity. The *y*-axis depicts the ratio between observed and expected number of overlaps (based on 10,000 randomic simulations) between CNV breakpoints and homologous regions. Homologous regions are placed in one of the bin classes in the *x*-axis which are based on inter- or intrachromosomal percent identities. Permutation *p*-values are based on the number of random simulations that obtained more overlaps than observed (* ≤ 0.05 and *** ≤ 0.001)

It has been shown that homologous regions reflect segmental duplications and promote CNV formation [43]. In order to study this in great tits we identified large homologous regions (≥ 1 kb and at least 90% sequence identity) using megablast [44]. We identified 3.44Mb of the automosomes as homologous regions (0.37%), representing 1,111 intra- and 879 inter-chromosomal homologies respectively (Table 1). The breakpoints observed at very low frequency (≤ 2) are not correlated with the occurrence of homologous sequences whereas the more frequent ones (>3) show progressively more overlap with homologous regions (Fig. 3a). The sequence identity of the homologies is also correlated with breakpoint frequency. Homologous regions with higher sequences identity tend to overlap more with CNV breakpoints with a frequency equal or more than four (Fig. 4), in agreement with previous studies in human and chimpanzee describing an excess of CNVs at regions with high sequence homologies [12].

**Table 1:** Homologous regions in the great tit genome with more than 90% of sequence identity and respective proportions of intra and interchromosomal homologies

Homology	Number of regions	Total size (Mb)	Similarity (+-SD)
Intrachromosomal	1111	2.66	92.97+-2.26
Interchromosomal	879	1.58	92.78+-2.1
All	1512	3.44	92.89+-2.25

In addition to the homologous regions, we identified repetitive elements masked by RepeatMasker [45]. These elements represent 6.16% (56.92 Mb) of the total length of the great tit autosomes. We found 400,503 masked regions, representing mainly retroelements (145,689; 43.06 Mb), *in-tandem* repeats (240,115; 11.54Mb) and DNA transposons (13,374; 1.95 Mb; all regions and sub-classification are shown in [Additional file 4]). All frequencies of CNV breakpoints (Fig. 2) overlap masked regions more than expected by chance, but there was no correlation between the overlap and frequency (correlation coefficient = 0.16, *p*-value = 0.66, Fig. 3b).

Noteworthy is that although homologous and masked regions show substantial overlap, their distribution differs. Intervals covered by both features (i.e. intersection) are considerably smaller than the regions overlapped in each of them. From 1,512 homologous regions, 1,302 (3.13 Mb; 91%) overlap intervals masked by RepeatMasker [45] by at least 1 bp. From 397,537 masked regions, 2,594 (1.24 Mb; 2.18%) overlap homologous regions by at least 1 bp. However, only 985 kb is covered by both (31.5% and 1.73% of the total length in homologous and masked regions respectively).

**Fig. 5:**
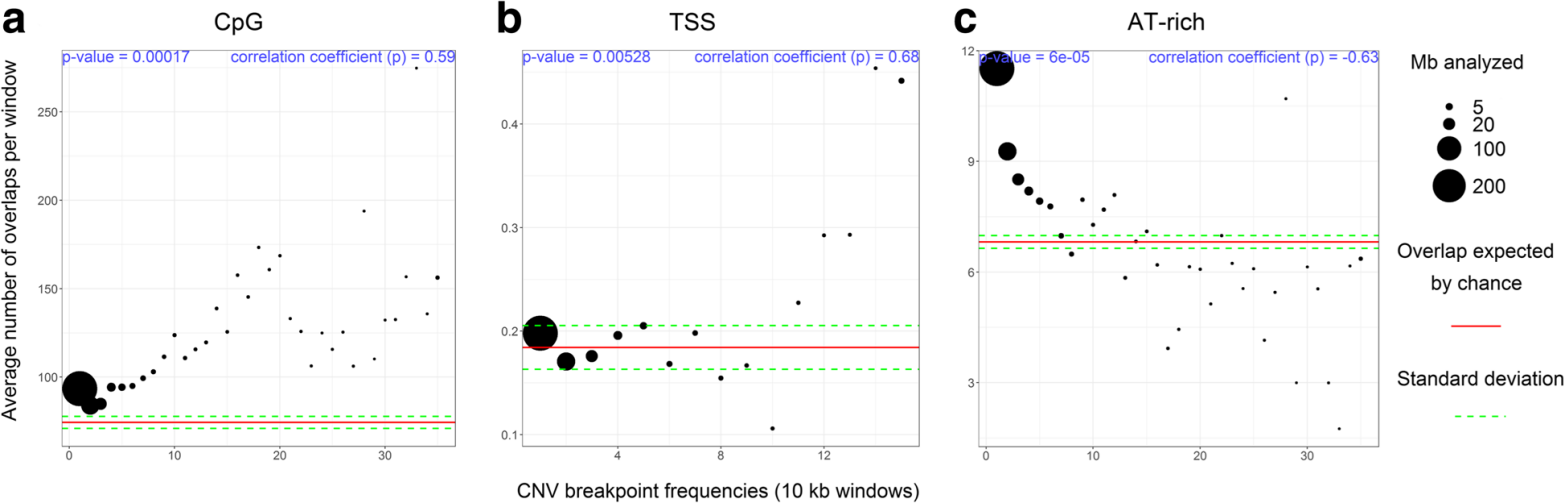
Overlap of CNV breakpoints with functional features and regions prone to breakage. **a**: CpG sites. **b**: Transcription start sites (TSSs). **c**: AT-rich intervals. CNV breakpoints observed in 30 birds or more are grouped together for CpG and AT-rich features. Otherwise, in TSSs we grouped those with 10 or in frequency because most of high frequent CNV breakpoints are small groups and can impair confident comparison with more scarce features as TSSs (in comparison with CpG or AT-rich sites)

Genomic regions which are rich in CpG sites and TSSs show a high recombination rate in birds [32]. Thus, we inferred these two features to understand the association of highly recombinant regions with CNVs. We identified 6,861,240 CpG sites in the great tit autosomes, ranging from 12,725 on chromosome LGE22 to 845,266 on chromosome 2. All CNV breakpoints windows contain more CpG sites than expected by chance and the number of sites increases along with the breakpoint-frequency (correlation coefficient = 0.59, *p*-value = 0.00017, Fig. 5a). Similarly, TSSs have positive overlap correlation with CNV breakpoint frequencies (up to 50% of breakpoints with frequency ≥ 15 overlap with TSSs, Fig. 5b). Results from CpG sites and TSSs are expected to be comparable given the known high prevalence of CpG islands at TSSs [32, 46].

AT-rich intervals have been reported at genomic regions known to be prone to breakage, thereby allowing complex rearrangements [14]. Thus, we identified 629,840 AT-rich intervals, of which the majority is 8 bp in size but that can be up to 100 bp in size. CNV breakpoint frequencies have a strong negative correlation with AT-rich intervals (Fig. 5c).

To verify a possible technical bias underlying the observed correlations, we evaluated the correlation between signal variability in SNP probes outside our CNVRs and the GC ratio of the region. The GC ratio could be relevant because it can lead to a so-called GC wave [47], which is a well-known bias in the detection of CNVs from SNP-arrays (causing variation in hybridization intensity). We inferred the Log R Ratio (LRR) values in non-CNV probes and estimated its standard deviation median for each tile of 10 kb in the genome. We correlated these medians with the GC ratio and found a very low positive correlation coefficient (0.02; *p*-value=0.059) with the LRR standard deviation (SD) median [Additional file 5]. This low correlation is expected because we corrected all LRR values for this GC wave before CNV detection.

### Gene enrichment and functional analysis

The genomic coordinates of all 8,008 CNVRs identified overlap with 6,857 of the 16,541 annotated unique genes (41.45%) for great tit (build 1.1 [40]). Using these overlapping genes we performed an enrichment analysis looking for pathways (Kyoto encyclopedia of genes and genomes, KEGG) and gene ontology (GO) gene sets prevailing in genes located within (i) CNVRs and (ii) CNV breakpoints seen in at least four birds.

Proteins of genes overlapping CNVRs were significantly overrepresented for 15 KEGG biological pathways (Table 2, [Additional file 6]), which are mostly related to neuronal and cardiac processes. All significant KEGG pathways were compared with 10,000 random enrichments and we found all processes enriched in CNVRs with permutation *p*-value ≤ 0.001 [Additional file 6]. In accordance with KEGG results, we found 77 GO gene sets mostly related with neuronal, cardiac and ion transport pathways. The GO gene sets with lowest *p*-values where synaptic membrane, postsynapse and postsynaptic membrane respectively [Additional file 6].

**Table 2:** Biological pathways enriched at CNVRs in the great tit genome

ID	Description	Number of proteins	Ajusted *p*-value	Protein ratio
hsa05412	Arrhythmogenic right ventricular cardiomyopathy (ARVC)	59	5.15 × 10^−6^	0.728
hsa04020	Calcium signaling pathway	126	1.16 × 10^−4^	0.583
hsa04360	Axon guidance	127	3.99 × 10^−4^	0.57
hsa04724	Glutamatergic synapse	78	8.2 × 10^−4^	0.609
hsa04514	Cell adhesion molecules (CAMs)	75	8.2 × 10^−4^	0.638
hsa04925	Aldosterone synthesis and secretion	60	8.2 × 10^−4^	0.61
hsa04713	Circadian entrainment	67	3.1 × 10^−3^	0.604
hsa00220	Arginine biosynthesis	19	3.15 × 10^−3^	0.826
hsa04970	Salivary secretion	48	1.34 × 10^−2^	0.615
hsa04022	cGMP-PKG signaling pathway	105	1.73 × 10^−2^	0.591
hsa05410	Hypertrophic cardiomyopathy (HCM)	55	1.73 × 10^−2^	0.536
hsa04740	Olfactory transduction	29	1.73 × 10^−2^	0.674
hsa05010	Alzheimer’s disease	78	3.84 × 10^−2^	0.545
hsa04750	Inflammatory mediator regulation of TRP channels	60	4.92 × 10^−2^	0.561
hsa05414	Dilated cardiomyopathy	57	4.92 × 10^−2^	0.564

ID = pathway identification code; Description = pathway name; Number of proteins = number of protein names with genes overlapping CNVRs; Adjusted *p*-value = enrichment FDR corrected *p*-value; Protein ratio = ratio between protein names with genes in CNVRs and all protein names assigned to a specific pathway

In order to determine if similar enrichment is also reflected in more frequent CNVs, we performed the gene enrichment using the CNV breakpoint windows (frequency ≥ 4, subset analyzed in the Fig. 4). These CNV breakpoints overlap 1,012 genes which are enriched for five KEGG pathways and six GO gene sets, as presynaptic active zone, homophilic cell adhesion and neuron recognition [Additional file 7]. From these 1,012 genes, a subset of 68 overlap homologous regions in the great tit genome, 18 have SNP alleles previously described as under selection [40] and five overlap homologous regions and are under selection concomitantly [Additional file 10].

### Genome Synteny with zebra finch and chicken at great tit CNVRs

We compared the great tit genome with the genomes of chicken and zebra finch to identify synteny blocks [Additional file 8]. For the great tit-chicken comparison, we found 13,437 blocks in synteny ranging in size from 181 bp to 2.15 Mb. The number of blocks varied from 11 on chromosome LGE22 to 1,921 on chromosome 2. For the great tit-zebra finch comparison, we found 5,141 synteny blocks ranging in size from 182 bp to 6.19 Mb. The number of blocks varies from 18 on chromosome LGE22 to 605 on chromosome 2.

We then inferred to what extent the identified CNVs overlap with evolutionary breakpoints and whether this overlap differs from overlap with regions randomly chosen within the genome. We found 3,090 CNVRs (38.58%) overlapping evolutionary breakpoints (with chicken and zebra finch concomitantly), a number that is consistently higher than expected by chance (*p*-value 9.99e-05). We observed 7,022 genes overlapping the evolutionary breakpoints, which are enriched for biological pathways mostly related to neuronal and cardiac processes [Additional file 9]. At least eight genes that have previously been reported [48] to be located at CNV regions in chicken and four in zebra finch overlap evolutionary breakpoints [Additional file 10].

## Discussion

Most studies have focused on single nucleotide changes when studying genetic associations with phenotypes and evolution. However, also variation in genomic structures such as CNVs are shown to be associated with a wide range of phenotypes [19, 49] and evolutionary phenomena like speciation [12, 21, 50] and adaptation [51, 52]. We here therefore used a high density SNP array to identify CNVs as well as their inheritance and architecture in the great tit genome. We detected CNVs covering a large percentage (28.09%) of the great tit genome. Because CNV identification based on SNP Affymetrix arrays are prone to high false discovery rates, we used the mother-daughter family structure of our data to access relative CNV confidence. The relative number of inherited events is higher for CNVs supported by more SNP probes, especially for CNVs with more than 40 probes. The low inheritance of the shorter CNVs suggests a relative high false negative call rate. On the other hand, most of the CNVs tested by qPCR were successfully validated (15/16) and all of these had less than 25 probes suggesting a low false positive call rate of the Affymetrix array. Regarding the exact number of copies, the disparity between SNP-array and qPCR results can be explained by the inherent resolution of each technology. SNP-array data have limited power to infer the exact number of copies whereas qPCR may be considered a gold standard and consequently is more reliable to infer the number of copies.

We evaluated the overlap pattern of CNVs with five genomic features that have known role in structural variation formation and recombination: (i) Homologous regions, or segmental duplications, which support CNV formation through non-allelic homologous recombination [29, 53]. (ii) Repetitive features like transposable elements and retrotransposons which account for a substantial fraction of copy-number differences [54, 55] and mutually explain recent and ongoing phenotypic adaptation [56]. (iii) Functional CpG and (iv) TSSs that harbor high recombination rate in birds [32]. (v) AT-rich regions are prone to break and subsequently produce complex rearrangements [14, 29, 57–59]. All these five genomic features display non-random overlap with CNVs and their breakpoint frequencies.

Homologous regions, at least one kb in size and with at least 90% of sequence identity, reflect recent segmental duplications in the genome [43] and can increase the chance of a triplication event in subsequent generations by more than 100-fold [60]. Thus, apart from positive selection or drift, the CNV frequency may have increased due to a higher rate of rearrangement at these genomic intervals. We find a significant positive correlation between, CNV breakpoints seen in at least four birds, and regions containing segmental duplications. How similar these genomic homologies are, is also determinant for CNV formation and can reveal its evolutionary history [12]. Over time, duplicated regions that are fixed decrease in identity, which consequently decreases the chance of recombination mechanisms, such as non-allelic homologous recombination, to act upon them [61]. Therefore, CNVs arising from this mechanism are relatively rarer at duplications with lower homology. This is reflected by the increasingly overlap of CNV breakpoints (frequency ≥ 4) and homologous regions with higher sequence identity.

Most of homologous regions overlap repetitive elements masked in the genome, like transposable elements. However, both features display different genomic length distribution and coverage. Repetitive elements cover around ten times more nucleotides, but are usually smaller in length when compared with overlapping homologous regions. In addition, masked regions overlap CNV breakpoint windows more than expected by chance but do not differ between breakpoint frequencies like homologous regions. The number of transposable elements in the great tit genome is comparable with other bird genomes, but they cover a relatively smaller fraction of the whole genome sequence length. The relative coverage in great tit is 1.24% whereas other bird species vary from 4.1 to 9.8% ([62–64], for a review see [65]). The coverage of transposable elements found here for the build 1.1 is comparable to previous version of the genome (2.06 Mb in this study and 1.95 Mb previously in [40]). Remarkably, transposable elements in great tit genome display distinct CpG hypermethylation between tissues, albeit their expression is correlated only with non-CpG methylation [46].

We also evaluated whether the CNV breakpoints are positively correlated with the presence of functional sequences like CpG sites and TSS. It has been shown that in birds recombination prevails at transcription start or end sites and CpG islands [32]. The overlap of CpG sites and TSSs with CNV breakpoints increases with breakpoint frequencies in this great tit population. This result suggests a higher CNV mutation rate at these regions, although it is complex to disentangle mutation rate from selection of the CNVs at these regions.

AT-rich intervals have repeatedly been reported as common fragile sites [29, 57, 58], which are more prone to break induced replication [66]. This mechanism has a high risk of undergoing template switching [14, 59], resulting in complex structural variants. Therefore, as AT-rich intervals are expected to easily break during meiosis, each meiosis breakage might produce CNVs with distinct breakpoints and gene content in the population [29]. CNV breakpoint frequencies in this great tit population are negatively correlated with AT-rich sites, in agreement with the expectancy that lower number of CNVs will share breakpoint positions among individuals in fragile sites throughout genome.

We also performed a functional enrichment for genes within (i) CNVRs and (ii) CNV breakpoints seen in at least four birds. A large proportion of the great tit genes overlaps with CNVRs (41.76%) and these CNV breakpoints (6.12%). Although CNVRs overlap almost seven times more genes, pathways in CNVRs as well as in these CNV breakpoints were enriched to neuronal processes and structure like axion guidance and glutamatergic synapse; cardiac or muscular processes like arrhythmogenic right ventricular cardiomyopathy and calcium signaling. Interestingly, genes related to neuronal functions were previously shown to be under positive selection in great tit [40]. Moreover, a comparative CNV analysis among different bird species such as chicken, turkey and common quail found a gain in leucine rich repeat and fibronectin type III domain containing 5 (*LRFN5*), which is involved in presynaptic differentiation, to occur just in quails [67]. In this great tit population, *LRFN5* is located within CNVR7101 (frequency ≥ 5.4%) that harbor gains and losses. Calcium signaling, that is also enriched in great tit CNVRs, is a key process in neuronal physiology mainly due to its role on neuron buffering [68] and in muscle activity by troponin-tropomyosin complex ([69], for a review on calcium signaling see [70]). However, the high rate of false negative of the CNVs identified here hampered efforts to find which genes are under selection, or that display high LD with SNP alleles at genes previously found to be under selection [40].

We identified a median of 12 CNVs per bird, which is comparable to 11.75 found by Skinner et al. [67] that evaluated different bird species, which in turn is comparable to the situation in mammals [67]. The same study also claimed that CNVRs in birds could have a slightly higher association with genes than in mammals, but the limited number of samples prevented a more robust conclusion at that time. Here we found 66% of the CNVRs harboring genes, value that increases to 78.3% when considering only polymorphic CNVRs. These proportions are comparable with the 70% that has been found previously [67]. Therefore, the large population analyzed here plus the prevalence of bird CNVs on genes may explain the striking proportion of 41.45% great tit genes with CNVs.

To shed light on the evolutionary implications of CNVs and their associated genomic architecture, we compared the great tit genome with the genomes of two other birds: chicken and zebra finch. As expected, because of the higher evolutionary proximity we found a higher degree of synteny between the two songbirds, great tit and zebra finch. The overrepresentation of CNVs at evolutionary breakpoints suggests a critical role in speciation. Moreover, we found biological pathways that are related to neuronal and cardiac processes enriched in both CNVs and evolutionary breakpoints. Syntenic regions among zebra finch and chicken with known CNVs harbor at least nine genes that are at evolutionary breakpoints. These genes are involved in signalling and neuronal pathways.

## Conclusion

CNVs can be challenging to detect and interpret using SNP arrays due to biological and technical variability. The qPCR validation and the intrinsic genomic architecture of the CNVs identified here point to a substantial number of false negatives. The genomic features enriched in CNVs (homologous regions, masked regions, CpG sites, TSSs and AT-rich intervals) support specific mechanisms of the formation of CNVs. Moreover, CNVs are enriched at evolutionary breakpoints, neuron and cardiac related genes and a subset harbors SNP alleles under selection [40]. Therefore, we expect the CNVs identified here to be valuable for future studies on the great tit genome, but the non-random distribution and inheritance patterns of CNVs indicate that they should be interpreted in the light of their genomic architecture and false negative rate.

## Methods

### Genotype calling and population description

Blood samples of great tits (*Parus major*) were collected from our long-term study populations on the ‘Veluwe’ area near Arnhem (52°02’ N, 5°50’ E, the Netherlands). Whole blood samples were stored in either 1 ml Cell Lysis Solution (Gentra Puregene Kit, Qiagen, USA) or Queens buffer [71]. DNA was extracted by using the FavorPrep 96-Well Genomic DNA Extraction Kit (Favorgen Biotech corp.). DNA quality and DNA concentration were measured on a Nanodrop 2000 (Thermo Scientific).

A total of 2,648 great tits were genotyped using a custom made Affymetrix®;great tit 650K SNP chip at Edinburgh Genomics (Edinburgh, United Kingdom). SNP calling was done following the Affymetrix®;best practices workflow by using the Axiom®;Analysis Suite 1.1. Nine individuals with dish quality control value of <0.82 were discarded. The length of the probes is 70 bp and more information is available in the raw data submitted to gene expression omnibus (GEO, GSE105131).

### Input construction and individual CNV calling

We applied the files denominated ‘summary’, ‘calls’ and ‘confidences’, built during SNP genotyping, to obtain the inputs for CNV detection. These files were used to generate canonical clusters [72] by the PennCNV (version 08 Feb 2013) function ‘generate_affy_ geno_cluster.pl’, which allowed the estimation of the relative signal intensities (i.e. LRR) and relative allele frequencies (B allele frequency, BAF) by the ‘normalize_affy_geno_cluster.pl’ PennCNV function. Using individual BAF values we then estimated the population BAF for each SNP marker, with the ‘compile_pfb.pl’ PennCNV function.

As the CG ratio content around each SNP marker is known to influence the signal strength [47], their relative content (1 Mb window) was estimated using the ‘nuc’ BEDTools function [73]. Therefore, we used the ‘genomic_wave.pl’ PennCNV function to adjust individual raw LRR signal values.

To identify the individual CNVs, we applied the ‘detect_cnv.pl -test’ for all 31 autosomes. The raw CNVs were filtered out if smaller than 1 kb or supported by less than 3 SNPs. Birds with LRR standard deviation >0.30 or BAF drift >0.02 were also filtered out. A total of 2,175 birds had at least one CNV call after quality control.

### Establishment of CNV hotspots and CNV frequency

The genomic regions with at least one individual CNV mapped were defined by the ‘reduce’ function from GenomicRanges R/Bioconductor package (version 1.28, [74]) and then defined as CNVRs. The frequency of each CNVR was estimated based on the number of samples mapped at the genomic interval comprised by the CNVR.

We inferred the frequency of all CNV start and end positions and extend by 5 kb up and downstream these breakpoints. These genomic intervals are defined throughout the text as CNV breakpoint windows and their coordinates were compared with functional and repetitive intervals in the great tit genome.

### CNV validation by quantitative PCR

Primers were designed using Primer3plus [75] and quality testing was performed with NetPrimer (http://www.premierbiosoft.com/netprimer).

Samples to be validated were checked for quality based on the amount of dsDNA, which was measured with Qubit®;Fluorometer. Subsequently, in each sample we used four different concentrations to determine primer efficiency: 15ng, 7.5ng, 3.8ng and 1.9ng of DNA. Reactions were joined in a final volume of 12.5 *μ*l, containing 3.75 *μ*l DNA, 6.25 *μ*l 2X reaction buffer (MESA Blue from Invitrogen®;), 1.25 *μ*l forward primer (2 *μ*M) and 1.25 *μ*l reverse primer (2 *μ*M). Samples with CNV and diploid (2n, reference samples) were tested with the designed primer sets. Measurements were performed with the Applied Biosystems®;7500 real-time PCR system. Cycle thresholds (log2 Ct) were corrected based on the efficiency of each primer. *Δ*Ct was calculated as Ct from the sample with a specific CNV minus Ct of the diploid (2n) reference sample [76]. The reference sample was given by a random bird with 2n state on the tested region.

### Identification of repetitive regions in the great tit genome

To identify masked regions in the reference genome and their respective functionality we applied RepeatMasker [45] version open-4.0.6 using the default mode run with cross match version 0.990329. The query species was assumed to be ‘aves’. The regions identified were classified as retroelements, RNA-related regions, DNA transposons and *in-tandem repeats*. Subclassification to define the families within each class was also described when available for a specific class. For simplification, we considered three general families in retrotransposons (short interspersed nuclear elements [SINEs], long interspersed nuclear elements [LINEs] and long terminal repeats [LTRs]) and *in-tandem* repeats (satellites, regions of low complexity and simple repeats). Uncertain family classification was neglected in DNA transposons (e.g. “hAT?” was considered “hAT”).

To identify homologous regions in the great tit genome we used a protocol described elsewhere [77], which applied the megablast greedy algorithm [44] on the great tit reference genome build 1.1, [40]. We performed all possible comparisons among autosomes and each one against itself to identify inter and intra chromosomal duplications, respectively. We subset regions larger than 1 kb and >90% in sequence similarity, which suggest regions containing recent segmental duplications [77]. We filtered out all homologies with more than 10% of its size containing unknown nucleotides (“N”) or/and with less than 1 kb of know nucleotides: adenine (A), cytosine (C), thymine (T) or guanine (G).

### Functional features and patterns in great tit genome

Thus, we identified genomic intervals containing [*CG*]_*n*_ (_*n*_ = 1) and TSSs (defined the gene promoters as regions starting 300 bp upstream and ending 50 bp downstream each gene start position, always considering the transcription orientation in each gene). We also identified regions rich in AT ([*AT*/*TA*]_*n*_ or [*AA*/*TT*]_*n*_, where _*n*_≥ 4), due to their role on recombination by break induced replication [66]. CpG sites and AT-rich intervals were converted into reference genomic ranges (build 1.1 [40]) by ‘vmatchPattern’ function in GenomicRanges Bioconductor/R package (version 1.28, [74]). The overlap expected by chance was obtained by simulating genomic tiles of 10 kb with ‘randomizeRegions’ function in regioneR R/Bioconductor package (version 1.80, [78]).

### Gene annotation and enrichment analysis

We used gene annotation version 101 from the general feature format (GFF) file from National Center for Biotechnology Information (NCBI) great tit genome 1.1 (https://www.ncbi.nlm.nih.gov/assembly/GCF_001522545.2). From 17,545 unique gene names, 16,541 were assigned to autosomal chromosomes which were then used to the subsequent enrichment steps. Gene names were converted to Entrez Ids and subsequently enriched with ‘enrichKEGG’ function to identify KEGG pathways; and ‘enrichGO’ function to identify GO gene sets overrepresented in all CNVRs and in CNV breakpoint windows present in four birds or more. Both functions, implemented in the *ClusterProfiler* R/Bioconductor package (version 3.4.1, [79]), used human as the organism (org.Hs.eg.db R/Bioconductor package version 3.4.1, 2017-Mar29, [80]) due to high accuracy in gene and pathway annotation. The *p*-values were adjusted by Benjamini and Hochberg method (FDR [81]). The gene background to enrichment of CNV breakpoint windows included just genes up to 5 kb from SNPs (reflecting every 10 kb window around SNPs).

To infer the enrichment expected by chance using the same number of genes, we randomly sampled 6,812 genes (total number of unique gene names overlapping CNVRs) 10,000 times and followed the same enrichment process. Thus, for each significant KEGG pathway in CNVRs, we compared the number of protein/gene names in CNVRs with random enrichments. Therefore, the permutation *p*-value was based in the number of times that a random enrichment obtained equal more protein/gene names linked to a specific process (times/10,000).

### Identification of Syntenic blocks and evolutionary breakpoints

We used the chicken (*Gallus gallus*, Gallus_gallus-5.0) and zebra finch (*Taeniopygia guttata*, taeGut3.2.4) genomes to find sequence synteny with the great tit genome build 1.1 [40]. All FASTA files were used in the ‘FindSynteny’ and ‘AlignSynteny’ functions, which are both implemented in the R/Bioconductor package DECIPHER ([82], version 2.6.0). The synteny blocks were merged by overlap with ‘reduce’ function (GenomicRanges R/Bioconductor package, version 1.28, [74]). We classified the resulting output into (i) syntenic blocks, (ii) evolutionary breakpoints and (iii) evolutionary breakpoint regions as described previously [83].

## Additional files

Additional file 1 CNV regions (CNVRs) in the great tit (Parus major) genome. (CSV 333 kb)

Additional file 2 CNV breakpoints and their correspondent frequency. (TXT 1102 kb)

Additional file 3 qPCR validation of CNVs. Genomic regions validated, qPCR-PennCNV state correspondence and applied primers. (CSV 192 kb)

Additional file 4 Interspersed repeats and low complexity (regions masked by RepeatMasker [45]) and their family classification. (TXT 18544 kb)

Additional file 5 Correlation between GC ratio and the median of Log R Ratio (LRR) standard deviation in genomic tiles of 10 kb. (PDF 59 kb)

Additional file 6 Enrichment analysis of genes in CNVRs. (TXT 65 kb)

Additional file 7 Enrichment analysis of genes in CNV breakpoints (frequency ≥ 4). (TXT 3 kb)

Additional file 8 Syntenic regions in the great tit genome. (CSV 693 kb)

Additional file 9 Enrichment analysis of genes at evolutionary breakpoints of great tit in comparison with zebra finch and chicken. (CSV 633 kb)

Additional file 10 (i) Genes overlapping CNV breakpoints (frequency ≥ 4), homologous regions and SNPs under selection concomitantly; (ii) Genes overlapping evolutionary breakpoints at CNV regions in chicken and (iii) genes overlapping evolutionary breakpoints at CNV regions in zebra finch. (CVS 696 kb)

## Abbreviations

A: Adenine
AT: Adenine and thymine
bp: Base pair
C: Cytosine
CNV: Copy number variation
CNVR: Copy number variation region
CpG: Cytosine phosphodiester-bond guanine
G: Guanine
GC: Guanine and cytosine
GEO: Gene expression omnibus
GFF: General feature format
GO: Gene ontology
kb: Kilobase
KEGG: Kyoto encyclopedia of genes and genomes
LINEs: Long interspersed nuclear elements
*LRFN5*: Leucine rich repeat and fibronectin type III domain containing 5
LRR: Log R ratio
LTRs: Long terminal repeats
Mb: Millions of base pairs
NCBI: National center for biotechnology information
qPCR: Quantitative polymerase chain reaction
SD: Standard deviation
SINEs: Short interspersed nuclear elements
SNP: Single nucleotide polymorphism
SOX5: SRY-Box 5
T: Thymine
TSS: Transcription start site
