# Supplementary figures and images for: CNVs are associated with genomic architecture in a songbird
Source: BMC Genomics. 2018 Mar 13;19:195. doi: 10.1186/s12864-018-4577-1 (PMC6389189; doi:10.1186/s12864-018-4577-1)

## GC content

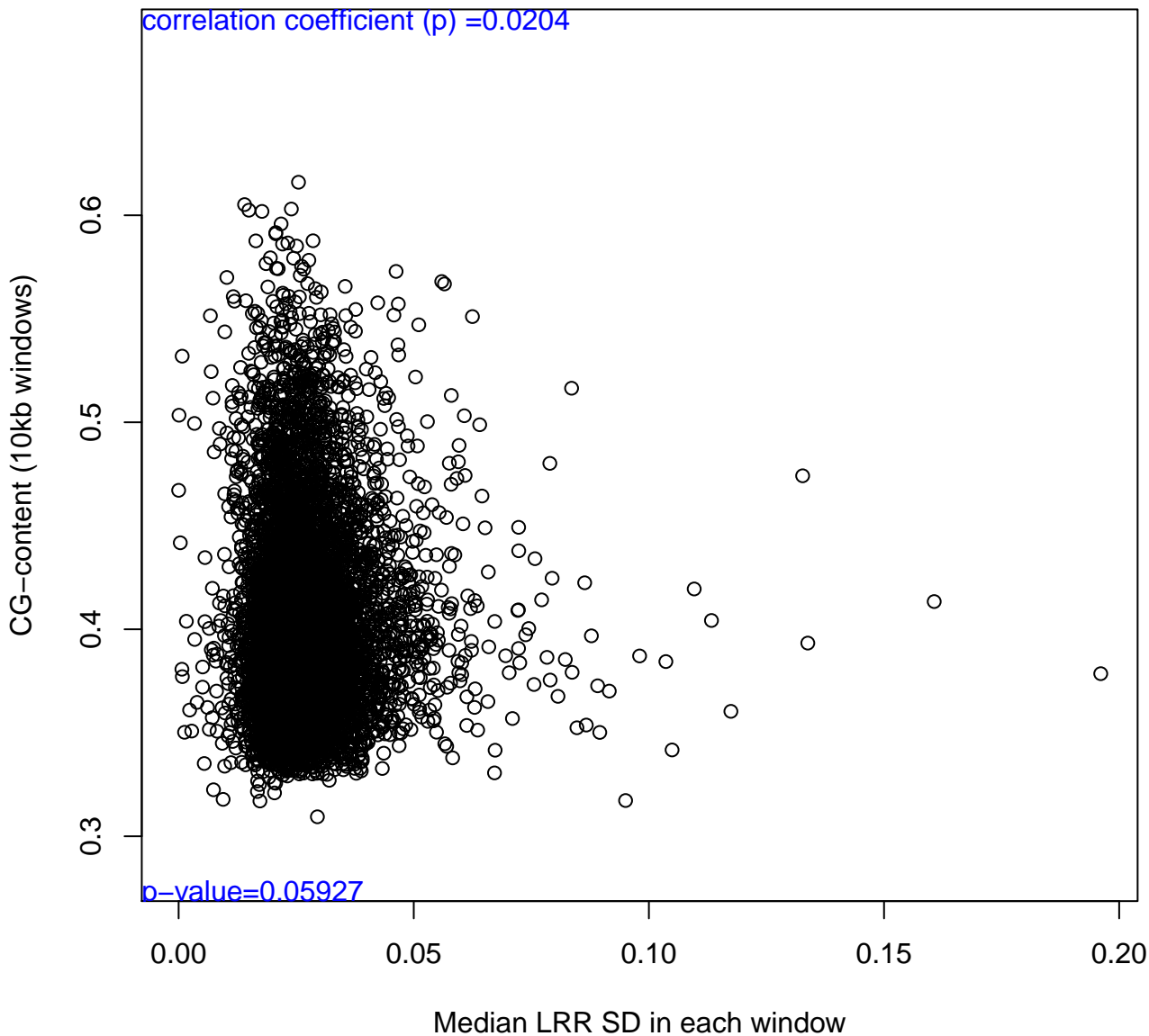

Supplement: Supplementary file 5 — Correlation between GC ratio and the median of Log R Ratio (LRR) standard deviation in genomic tiles of 10 kb. (PDF 59 kb) [file 12864_2018_4577_MOESM5_ESM.pdf]
